# Maintenance Low-Dose BRAF Inhibition and Rituximab in Relapsed Hairy Cell Leukemia: A Therapeutic Alternative

**DOI:** 10.7759/cureus.95668

**Published:** 2025-10-29

**Authors:** Anshul Patel, Sarina Koilpillai, Cassie Nguyen, Said Baidas

**Affiliations:** 1 Hematology and Medical Oncology, Orlando Health Cancer Institute, Orlando, USA; 2 Clinical Pharmacy Specialist/Oncology Hematology Services, Orlando Health Cancer Institute, Orlando, USA

**Keywords:** anti-cd20 monoclonal antibody, braf inhibitors, hairy cell leukemia (hcl), hairy cell leukemia relapse, vemurafenib

## Abstract

Hairy cell leukemia (HCL) is a rare, indolent B-cell neoplasm accounting for less than 2% of all leukemias. It is characterized by the presence of mature lymphocytes with typical hairy projections within the peripheral blood, bone marrow, and spleen, resulting in pancytopenia and splenomegaly. Purine analogs such as cladribine and pentostatin have been the mainstay of treatment for HCL. Despite high responsiveness to first-line therapy with purine analogs, almost half of the patients with HCL relapse and become progressively resistant to these medications. Treatment with a combination of BRAF V600E inhibitor and anti-CD20 monoclonal antibody has achieved durable responses in relapsed HCL. Here, we present a patient with relapsed and refractory HCL who achieved only partial response with three months of treatment with high-dose vemurafenib and rituximab, following which he was maintained on low-dose vemurafenib plus monthly rituximab for almost a year, after which he achieved complete morphologic and molecular response.

## Introduction

Hairy cell leukemia (HCL) is a rare, indolent B-cell neoplasm accounting for less than 2% of all leukemias [1]. It is characterized by the presence of mature lymphocytes with typical hairy projections within the peripheral blood, bone marrow, and spleen, resulting in pancytopenia and splenomegaly. The initial treatment for HCL was splenectomy, which provided a good but short-lived response. Patients who relapsed were treated with low-dose chlorambucil. The five-year survival for all patients with this sequential approach was 68% [2]. The introduction of recombinant interferon alfa was first suggested in 1984 [3]. Interferon use showed partial responses and a six-year survival reaching up to 85% in some studies, although it was not curative [4]. In the 1980s, purine analogs were studied for the treatment of HCL. Pentostatin proved to be an effective therapy, achieving an overall response rate greater than 90% and a complete response (CR) rate ranging from 56% to 87% [5,6]. Treatment with cladribine reported CR rates of 90% with initial treatment, while subsequent treatment with cladribine for relapsed disease had CR rates around 60% [7,8]. Single-agent rituximab is a treatment option for HCL that has relapsed after purine analogs but results in a low rate of complete remissions [9]. Combination of rituximab and cladribine, however, improved response rates and durability. The overall response rate (ORR) was 87% with 61% achieving a complete response. The two-year PFS rate was 65% and the OS rate was 74%. For those subjects achieving a CR with a median follow-up of 23 months, only 5.3% had relapsed [10]. Hairy cells are strongly positive for CD22, which led to the development of moxetumomab pasudotox, an anti-CD22 immunotoxin conjugate [11]. However, due to serious side effects, it was discontinued by the manufacturer in 2023.

In recent years, enormous advances have been made in the understanding of HCL biology. Specifically, the BRAF V600E somatic mutation was found to be a characteristic molecular feature of the disease, present in over 95% of cases [12]. The mutation is responsible for continuous RAS-RAF-MEK-ERK pathway signaling, whose aberrant activation enhances the survival of the HCL cell [13]. Additionally, the BRAF V600E mutation remains stable throughout the entire disease course, from the initial diagnosis to relapse. Thus, BRAF-targeting agents offered a promising approach to the treatment of HCL. Vemurafenib at a dose of 960 mg twice daily for a minimum of 8 weeks, up to a maximum of 16 weeks, in two phase two studies achieved an OR of 91% with a CR of 35% [14]. Originally, the dosing and scheduling of BRAF inhibitors were extrapolated from melanoma studies. Based on the retrospective analyses, BRAF inhibitors induce hematologic response in HCL patients, regardless of dosage [15]. However, higher doses of BRAF inhibitors (vemurafenib greater than 480 mg twice daily, dabrafenib greater than 150 mg twice daily) improve the quality of responses and prolong time to next treatment (TNT) when compared with low-dose BRAF inhibitors [16]. Notably, when BRAF inhibitors are the only available treatment option, treatment with low-dose BRAF inhibitors (less than 480 mg twice daily) showed stabilization of the disease for more than one year [16]. In this cohort, the treatment-free survival was longer in patients receiving high-dose BRAFi (greater than 480 mg twice daily) compared to those with low-dose BRAFi, but most patients were dosed intermittently to limit adverse effects, so a true comparison between low- and high-dose BRAF inhibition is lacking. A combination of BRAF inhibitors with monoclonal antibodies is an effective treatment for relapsed HCL. One study evaluated high-dose vemurafenib 960 mg twice daily and eight doses of rituximab 375 mg/m2 over 18 weeks in refractory HCL after purine analogs. This resulted in a CR rate 87% with 17% achieving MRD negativity [17]. Progression-free survival was 78% at 37 months, and relapse-free survival was 85% at a median follow-up of 34 months. This study showed that deeper and longer responses can be achieved when rituximab is combined with vemurafenib. This study also claimed that lower doses of vemurafenib were associated with relapses or lack of response. Vemurafenib was also combined with Obinutuzumab in previously untreated HCL, and all patients remained in remission at 9.7 months [18]. Ultimately, combination therapy of BRAF inhibitors and CD20 targeting agents can improve the depth and duration of response in HCL that has relapsed after treatment with purine analogs.

Here, we present a patient with relapsed and refractory HCL who achieved only a partial response with three months of treatment with high-dose vemurafenib and rituximab. Subsequently, he was maintained on low-dose vemurafenib plus monthly rituximab for almost a year, after which he achieved complete morphologic and molecular response.

## Case presentation

A 71-year-old male was initially diagnosed with HCL in 1982 and was initially treated with splenectomy. He had relapsed disease in the early 1990s and was treated with pentostatin. A second relapse occurred in early 2000, and he was once again treated with pentostatin. After this, he was disease-free for 20 years. In October 2020, a complete blood count (CBC) showed significant leukocytosis. A bone marrow biopsy revealed HCL, and peripheral blood Next-Generation Sequencing (NGS) detected a BRAF V600E mutation. The patient was treated with cladribine and rituximab, and a post-treatment bone marrow biopsy was negative for residual HCL. 

**Figure 1:**
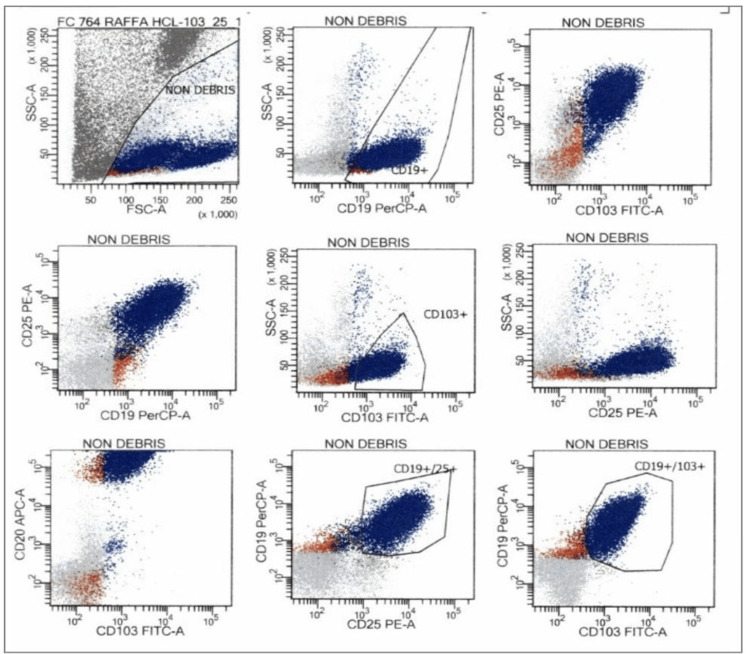
Flow cytometry positive for CD103, CD25, and CD10, consistent with hairy cell leukemia.

He was in remission until August 2022, when a CBC reported white blood cell count increased to 17,000/uL. A peripheral blood smear revealed 78% atypical lymphocytes, 11% mature lymphocytes, an absolute neutrophil count of 1,000/µL, a platelet count of 240,000/µL, and a hemoglobin level of 14.1 g/dL. A peripheral blood flow cytometry was positive for CD11c, CD19, CD20, CD22, CD25, CD38, CD45, CD103, HLA-DR, and surface kappa, confirming HCL (Figure 1).

**Figure 2:**
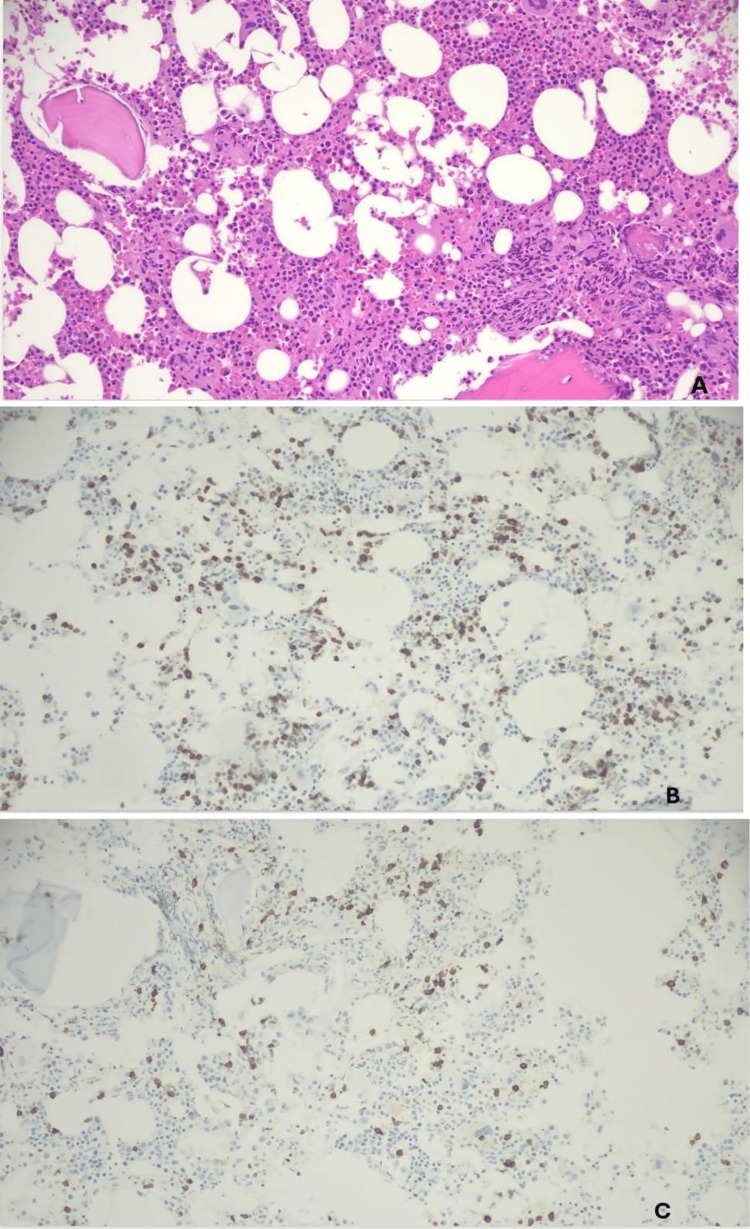
Bone marrow core biopsy. Residual hairy cell leukemia with 10%-20% leukemic cells and 40% cellularity. Trilineage hematopoiesis is preserved. (A) 20× H&E stain; (B) 20× CD79a stain; (C) 20× CD3 stain.

By November 2022, his white blood cell count had risen to 50,000/µL with over 90% variant lymphocytes, hemoglobin 13.4 g/dL, and platelets 167,000/µL. Since he previously received two courses of pentostatin and relapsed within two years of receiving cladribine, it was unlikely that repeating either treatment regimen would result in a meaningful remission. It was therefore recommended to start therapy with BRAF inhibitor vemurafenib and anti-CD20 monoclonal antibody rituximab. Each 42-day cycle consisted of vemurafenib 960 mg twice daily for 28 days followed by 14 days off, along with rituximab 375 mg/m² on days 1 and 15. He completed three months of treatment consisting of two cycles of vemurafenib and eight doses of rituximab given every two weeks. He tolerated treatment well aside from grade 1 fatigue and skin tags, with pathology revealing keratoacanthoma. In March 2023, after three months of high-dose vemurafenib and rituximab, his CBC was normal with white blood cell count 5,400/µL with hemoglobin 13.7 g/dL, and platelets 293,000/µL. Differential count showed absolute neutrophil count 3,500/µL and absolute lymphocytes at 700/µL (16.1%) with no atypical lymphocytes, and BRAF mutational testing by Sanger sequencing was negative in peripheral blood. A bone marrow biopsy showed a 40% cellularity with trilineage hematopoiesis and revealed residual disease with 10%-20% HCL. BRAF mutational testing by Sanger sequencing was positive (Figure 2).

**Figure 3:**
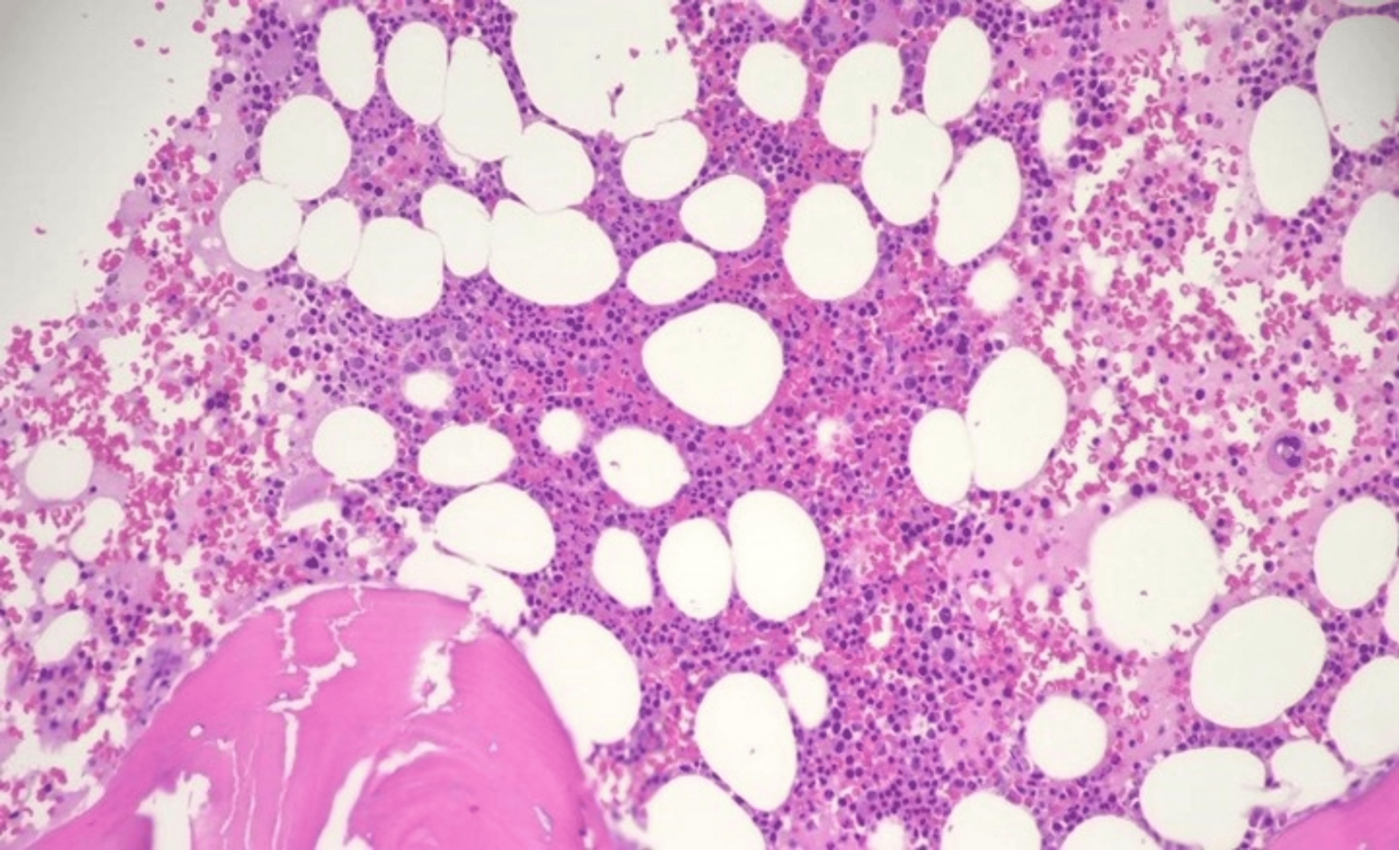
Bone marrow core biopsy (20× H&E stain). Normocellular marrow (~40%) with trilineage hematopoiesis. No evidence of hairy cell leukemia.

Given this partial treatment response, it was decided to transition the patient to a maintenance dose of vemurafenib 240 mg twice daily and rituximab 375mg/m2 every four weeks. He remained on maintenance treatment for 10 months. His CBC was normal. A repeat bone marrow biopsy after completion of maintenance therapy (Figure 3) revealed normocellular marrow (40% cellularity) with trilineage hematopoiesis and no morphological evidence of HCL. The BRAF V600E mutation was no longer detected by Sanger sequencing. He has now been off treatment for more than a year and continues to be in remission.

Images are obtained from the Division of Pathology at Orlando Health.

## Discussion

Although we have evidence of excellent response rates with high-dose BRAF therapy, this treatment is not feasible for a prolonged period due to intolerance and toxicity. Low-dose vemurafenib as single-agent therapy for HCL has reported suboptimal outcomes even with prolonged therapy. There are no studies exploring maintenance therapy with low-dose vemurafenib and concurrent rituximab for HCL. Our patient with relapsed, refractory HCL only achieved a partial response with three months of high-dose vemurafenib and rituximab. Therefore, we chose to start maintenance therapy with low-dose vemurafenib and monthly rituximab for almost a year, after which he achieved CR with minimal residual disease (MRD) negativity. He is now off treatment and continues to be in remission. Since our patient was unable to achieve a CR with every-2-weekly rituximab and high-dose BRAF inhibitor, but achieved MRD negativity by molecular testing with less frequent rituximab and low-dose BRAF inhibitor therapy, we pose the following questions:

Is prolonged therapy with low-dose BRAF inhibitors superior to short-term therapy with high-dose BRAF inhibitors? Will a low-dose BRAF inhibitor and rituximab every 28 days for one year have the same response as the initial three months of high-dose BRAF inhibitors? Should treatment of relapsed HCL be high-dose BRAF inhibitor and rituximab every 14 days for three months, followed by maintenance low-dose BRAF inhibitor and rituximab every 28 days for a total of one year? Should treatment of relapsed HCL be high-dose BRAF inhibitor and rituximab for one year?

We suggest a three-arm clinical trial. Arm A: Vemurafenib 960 mg twice daily and rituximab every two weeks for three months, followed by vemurafenib 240 mg twice daily and rituximab every four weeks for a total of 12 months. Arm B: Vemurafenib 960 mg twice daily for 12 months and rituximab every two weeks for three months, followed by monthly rituximab for a total of 12 months. Arm C: Vemurafenib 240 mg twice daily for 12 months and rituximab every two weeks for three months, followed by monthly rituximab for a total of 12 months.

## Conclusions

While prior studies have suggested that lower doses of vemurafenib are associated with poorer responses, low-dose vemurafenib with maintenance rituximab following induction therapy with high-dose BRAF inhibition provided a tolerable and effective long-term response in our patient. Overall, this case showed that in our patient with relapsed/refractory HCL, maintenance therapy with low-dose BRAF inhibitor and intermittent rituximab led to durable remission following partial response to induction therapy. It also highlights the need for a clinical trial to compare outcomes in patients with relapsed/refractory HCL being treated with BRAF inhibition and rituximab.

## References

[1] Teras LR, DeSantis CE, Cerhan JR, Morton LM, Jemal A, Flowers CR (2016). 2016 US lymphoid malignancy statistics by World Health Organization subtypes. CA Cancer J Clin.

[2] Golomb HM, Vardiman JW (1983). Response to splenectomy in 65 patients with hairy cell leukemia: an evaluation of spleen weight and bone marrow involvement. Blood.

[3] Flandrin G, Sigauz F, Castaigne S Treatment of hairy cell leukemia with recombinant -interferon: quantitative study of bone marrow changes during the first months of treatment. Blood.

[4] Spielberger RT, Mick R, Ratain MJ, Golomb HM (1994). Interferon treatment for hairy cell leukemia. An update on a cohort of 69 patients treated from 1983 to 1986. Leukemia Lymphoma.

[5] Grem JL, King SA, Cheson BD, Leyland-Jones B, Wittes RE (1989). Pentostatin in hairy cell leukemia: treatment by the special exception mechanism. J Natl Cancer Inst.

[6] Spiers AS, Moore D, Cassileth PA (1987). Remissions in hairy-cell leukemia with pentostatin (2'-deoxycoformycin). N Engl J Med.

[7] Konwalinka G, Schirmer M, Hilbe W (1995). Minimal residual disease in hairy-cell leukemia after treatment with 2-chlorodeoxyadenosine. Blood Cells Mol Dis.

[8] Pileri S, Sabattini E, Poggi S (1994). Bone marrow biopsy in hairy cell leukemia (HCL) patients. Histological and immunohistological analysis of 46 cases treated with different therapies. Leukemia Lymphoma.

[9] Nieva J, Bethel K, Saven A (2003). Phase 2 study of rituximab in the treatment of cladribine-failed patients with hairy cell leukemia. Blood.

[10] Spurgeon S, Pindyck T, Loriaux MM (2010). Cladribine plus rituximab is an effective therapy for newly diagnosed mantle cell lymphoma. Blood.

[11] Kreitman RJ, Dearden C, Zinzani PL (2018). Moxetumomab pasudotox in relapsed/refractory hairy cell leukemia. Leukemia.

[12] Tiacci E, Trifonov V, Schiavoni G (2011). BRAF mutations in hairy-cell leukemia. N Engl J Med.

[13] Oscier D, Stamatopoulos K, Mirandari A, Strefford J (2022). The genomics of hairy cell leukaemia and splenic diffuse red pulp lymphoma. Cancers (Basel).

[14] Tiacci E, Park JH, De Carolis L (2015). Targeting mutant BRAF in relapsed or refractory hairy-cell leukemia. N Engl J Med.

[15] Dietrich S, Pircher A, Endris V (2016). BRAF inhibition in hairy cell leukemia with low-dose vemurafenib. Blood.

[16] Liebers N, Roider T, Bohn JP (2020). BRAF inhibitor treatment in classic hairy cell leukemia: a long-term follow-up study of patients treated outside clinical trials. Leukemia.

[17] Tiacci E, De Carolis L, Simonetti E (2021). Vemurafenib plus rituximab in refractory or relapsed hairy-cell leukemia. N Engl J Med.

[18] Park JH, Shukla M, Salcedo JM (2019). First line chemo-free therapy with the BRAF inhibitor vemurafenib combined with obinutuzumab is effective in patients with HCL. Blood.

